# Molecular detection and genetic diversity of avian haemosporidian parasites in Iran

**DOI:** 10.1371/journal.pone.0206638

**Published:** 2018-11-09

**Authors:** Leila Nourani, Mansour Aliabadian, Omid Mirshamsi, Navid Dinparast Djadid

**Affiliations:** 1 Malaria and Vector Research Group (MVRG), Biotechnology Research Center (BRC), Pasteur Institute of Iran, Tehran, Iran; 2 Department of Biology, Faculty of Sciences, Ferdowsi University of Mashhad, Mashhad, Iran; 3 Research Department of Zoological Innovations, Institute of Applied Zoology, Faculty of Sciences, Ferdowsi University of Mashhad, Mashhad, Iran; Université Pierre et Marie Curie, FRANCE

## Abstract

**Background:**

The mobility of birds across or between continents exposes them to numerous vectors that have the potential to transmit pathogens and spread them into new regions. A combination of rich species diversity of birds along with the small amount of molecular studies in Iran makes observing the blood parasite distribution in wild avian populations indispensable for further estimation and administration of blood parasites.

**Methodology/Principal findings:**

In order to evaluate the infection rate and molecular context of avian blood parasites, bird samples were collected (passerine = 316 and non-passerine = 14) in eight provinces of northern Iran between June to September 2015 and 2016. All bird samples were examined for haematoprotozoan infections by morphological screening using light microscope and mtDNA *cytb* gene amplification. A total of 115 birds were positive for blood parasites by molecular approach (34.84% overall infection). The infection rate of *Haemoproteus*, *Plasmodium*, and *Leucocytozoon* were 33.03%, 1.21%, and 0.6%, respectively. Sequences analysis has detected 43 lineages in Iranian birds’ hosts. Lineages were attributed to three genera *Haemoproteus* (n = 37), *Plasmodium* (n = 4), and *Leucocytozoon* (n = 2), of which 23 lineages fully matched previously recorded sequences in GenBank and MalAvi data reciprocities. Five lineages of ACDUM1, ACDUM2, PARUS1, PYERY01, and SISKIN1 were detected in multiple hosts’ species from dissimilar families. In Bayesian tree, all sequences were clustered in three main monophyletic clades as *Haemoproteus*, *Plasmodium*, and *Leucocytozoon* genera.

**Conclusions/Significance:**

As the first study outlining the molecular detection of hematozoa of passerines from Iran, the current study has recorded 20 new lineages for three genera of *Haemoproteus*, *Plasmodium*, and *Leucocytozoon*. Additional investigations into these taxa in the avifauna for the other parts of Iran may provide extra information on blood parasites, hosts relationships and distribution patterns.

## Introduction

The avian apicomplexan species of *Plasmodium* Marchiafava et Celli 1885, *Leucocytozoon* Berestneff 1904 and *Haemoproteus* Kruse 1980 with an extensive range of vectors are the most frequently and worldwide distributed genera of blood parasites [1]. These protozoan taxa parasitize the vast majority of vertebrate hosts including birds, mammals, and reptiles [2]. These vector-borne pathogens, by enforcing significant ecological and evolutionary pressures on their hosts, are responsible for avian extinction and population decrease through the negative potential imapact on their fitness [3–8]. Although, hematozoa were taken into account as benign organisms with low pathogenicity in the wild, it has been established that critical infection by haemosporidians may lead to death, anemia, inflammation and other physiopathological conditions [3,9,10]. Birds with a high ability of movement may be exposed by numerous vectors which may elevate the potential risk of pathogen transmission by new lineages around the world [11]. Various haematophagous arthropods comprising mosquitoes (Culicidae), biting midges (Ceratopogonidae), Louse Flies (Hippoboscidae), and Black Flies (Simuliidae) are regarded as the main vectors of avian blood parasites [12]. Preceding studies have illustrated various infection rates between 0–100 percent in bird species around the world, based upon sampling area and detection procedures [13–16]. Previous morphological studies have proposed that the species of *Haemoproteus* are seemingly more host-specific than *Plasmodium*. The similar evolutionary history of a parasite species and its own host has been considered as the host-specifity [17]. Inversely, the presence of one species on different avian hosts has been mentioned as the host-shifts, which may cause the virulence alteration [18]. However, the level of vertebrate host specificity for most blood parasites’ species remains unknown [19].

To date, the literature has reported *Haemoproteus* spp. infection in various Iranian birds such as aquatic birds [20], passerines [21–23], and domestic birds [24,25]. Several studies have also listed the infection of avian species with *Plasmodium* spp. [26,27]. Furthermore, several species of birds have been reported as the infected hosts by *Leucocytozoon* spp. within the country [28]. The aforesaid studies were performed on morphological detection of avian blood parasites while this study is an investigation on the molecular detection of blood parasites of passerin from this region.

The current avian checklist has recorded more than 548 birds’ species from Iran, of which approximately 235 species belong to the passerines [29]. Due to the rich species diversity of birds and the very restricted number of molecular studies in this region, observing the blood parasites distribution in wild populations seems indispensable for further estimation and administration of blood parasites surveillance and control. In the present study, we amplified a 479 base pair (bp) fragment of mitochondrial gene *cytb* in haemosporidians’ genera from 72 individuals in Iran (i) to screen hematozoan parasites within Iranian birds, (ii) to assess whether birds within Iranian territories contain lineages previously not recorded from other regions, and (iii) to what degree a single host and/or host species may be infected by multiple parasite lineages.

## Materials and methods

### Ethical statement

This study was carried out in strict accordance with the recommendations in the guide for the care and use of animals for scientific purposes of the Ferdowsi University of Mashhad, Iran The protocol was approved by the committee on the Ethics of Animal Experiments of Ferdowsi University of Mashhad (protocol number: IR.MUM.FUM.REC.1397.035). Furthermore, all field works were approved by the Department of the Environment, which provided the authority and permission for sample collection from each location in this study (No: 93/61478). All birds were released after blood samples were collected, and all efforts were made to minimize their suffering.

### Collection of samples and microscopic examination

Blood samples were collected from wild birds (passerine = 316, and non-passerine = 14) in eight provinces form Iran comprising Razavi Khorasan, North Khorasan, Semnan, Golestan, Mazandaran, Gilan, Zanjan, and Ardabil between June to September 2015 and 2016. Birds were captured using mist nets and within minutes of capture, approximately 50–100 μl of whole blood was drawn from a brachial vein by insulin needles and was preserved into Queens’s buffer [30]. Two or three blood smears of each host were prepared in the field and fixed with absolute methanol. They were then stained with Giemsa and screened for infection of the above mentioned three genera. All smears were inspected using an Olympus BH2 light microscope provided with an Olympus DP7 digital camera and imaging software DP-SOFT, for 10–15 minutes at low magnification (× 400), and then at least 100 fields were examined under high magnification (× 1,000) with immersion oil [2].

### Extraction of genomic DNA, PCR, and sequencing

DNA extraction was performed on 330 blood samples using PrimePrep Genomic DNA Isolation Kit for blood” (GENETBIO Inc. Daejeon, South Korea) following manufacturer guidelines. All of the extracted DNA specimens were used for detection of parasite infection using—nested PCR approach. PCR amplifications were accomplished in 25μl volumes and included 50ng/μl of total genomic DNA, 1.5 mM MgCl_2_, PCR buffer 1X, 1.25 mM of each dNTPs, 0.6 mM of each primer, and 0.5 units of Taq DNA polymerase. The cycling programs for 25 and 35 cycles for outer and inner reactions with primers HaemFNI/HaemR3, HaemF/HaemR2, and HaemFL/HaemR2L were run for the parasites discovery using temperature profiles [5,31]. To determine the positive or negative samples, 2.5 μl of the final PCR product was run on 1% agarose gel. All reactions were performed along with negative (double-distilled H_2_O) and positive controls (infected specimens determined by microscopy screening) to evaluate the validity of the PCR and control for any other contaminations. Sequencing was performed by Macrogene Co. (Seoul, South Korea).

### Phylogenetic analysis

**Fig 1:**
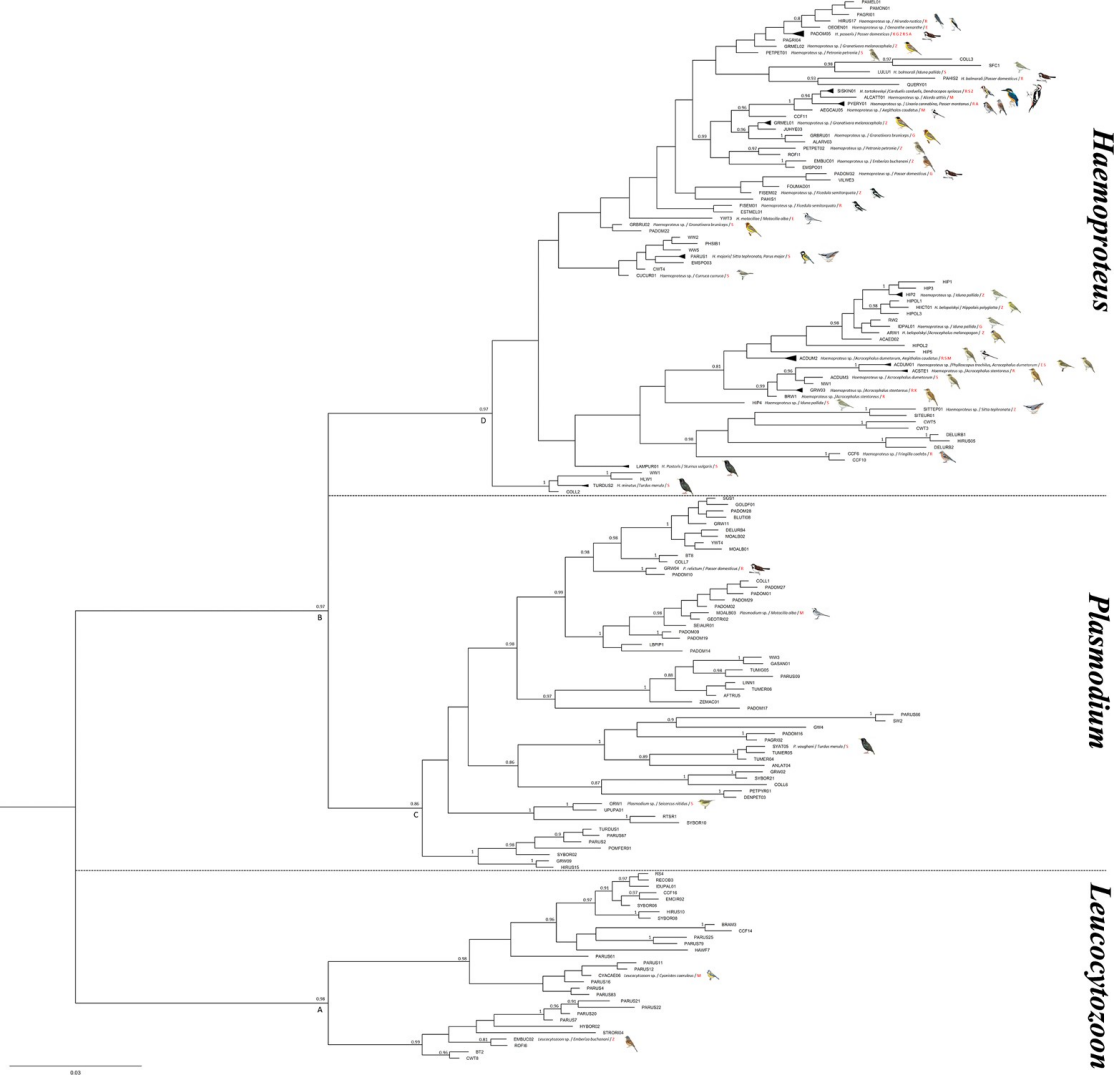
Bayesian phylogenetic tree of blood parasites of mitochondrial DNA *cytb* lineages attained from infected Iranian birds and MalAvi sequences. Posterior probability values (>0.8) are given. Provinces of sampling are abbreviated by Ardabil (A), Zanjan (Z), Semnan (E), North Khorasan (S), Razavi Khorasan (R), Golestan (G), Mazandaran (M) and Gilan (K). Schematic images of birds were retrieved from www.HBW.com.

The acquired sequences of 479 base pairs of *cytb* gene were edited, aligned and collated in a sequence identity matrix using BioEdit [32] and MAFFT online version [33]. All unique lineages were identified using theNational Center for Biotechnology Information (NCBI) Nucleotide BLAST search [34]. When comparing against the recorded dataset, sequences with one or more nucleotide substitutions were identified as new lineages according to the MalAvi Public Database [35]. Subsequently all sequences of the amplified lineages were deposited in both MalAvi database http://mbio-serv4.mbioekol.lu.se/avianmalaria [35] and GenBank (Accession numbers MG976505-MG976576). The sequence divergence between the different lineages was calculated with the use of a Kimura 2-parameter distance matrix, implemented in the program MEGA6.0 [36]. Phylogenetic relationships were estimated using Bayesian inference implemented in the program MrBayes v3.2 [37] using the sequence evolution model acquired from MODELTEST v3.7 [38]. MCMC analysis was run for 10,000,000 generations and sampled every 1,000 generations. At the end of the analysis, the burn-in period to 50% was set where the chains reached stationary status to calculate the posterior probabilities. The phylogenetic tree was constructed using FigTree v1.3.1 [39]. A resultant Bayesian analysis tree showed lineages situated within three main genera with bootstrap support of each cluster in addition to previously published lineages for similar hosts species around the world (Fig 1).

## Results

### Hosts sampling

In order to detect haemosporidian parasites, 330 birds were examined. Among well-sampled hosts (i.e. > 5) individuals were captured per species. The inspected population consisted of 316 individuals belonging to 37 species, 31 genera and 16 families of passerine and 14 individuals belonging to five species, five genera and five families of non-passerine birds. Family Passeridae was the most frequently captured group (28.79%) and *Passer domesticus* was the most captured host species. The highest percentage of collected samples was recorded in Razavi Khorasan province (27.27%) and the lowest percentage in Semnan (1.81%) province (Table 1).

**Table 1:** The identified lineages of blood parasites are illustrated for each host species, host family and location of sampling. The frequency of infected hosts for *Haemoproteus*, *Plasmodium*, and *Leucocytozoon* using PCR detection of blood parasites from Iran is shown in bracket.

Host family	Host species	Movements	Host frequency	*Haemoproteus*	*Plasmodium*	*Leucocytozoon*
Acrocephalidae	*Acrocephalus dumetorum*	W R	13	ACDUM1 (1) ^∞^ ACDUM2 (6) ^∞^ ACDUM3 (1)	0	0
	*A*. *melanopogone*	R	5	ARW1 (1)	0	0
	*A*. *stentoreus*	B	14	ACSTE1 (3) BRW1(3) GRW03 (3)	0	0
	*Iduna pallida*	B	24	HIP2 (4) IDPAL01 (1) ^¥^ HIP4 (2) LULU1 (3)	0	0
	*Hippolais polyglotta*	B	1	HIICT1 (1)	0	0
Aegithalidae	*Aegithalos caudatus*	R	8	ACDUM2 (1) ^∞^ AEGCAU05 (1) ^¥^	0	0
Alaudidae	*Galerida cristata*	R	1	0	0	0
Alcedinidae*	*Alcedo atthis*	B W P	6	ALCATT01 (1) ^¥^	0	0
Emberizidae	*Emberiza buchanani*	B	1	EMBUC01 (1) ^¥^	0	EMBUC02 (1) ^¥^
	*Granativora bruniceps*	B	16	GRBRU01 (1) ^¥^ GRBRU02 (3) ^¥^	0	0
	*G*. *melanocephala*	B P	15	GRMEL02 (2) ^¥^ GRMEL01 (4) ^¥^	0	0
Fringillidae	*Carduelis carduelis*	R	4	SISKIN01 (2) ^∞^	0	0
	*Erythrina erythrina*	P	1	0	0	0
	*Fringilla coelebs*	P W	5	CCF6 (1)	0	0
	*Linaria cannabina*	W R	14	PYERY01 (2) ^∞^	0	0
	*Serinus pusillus*	R	1	0	0	0
Hirundinidae	*Hirundo rupestris*	B	2	0	0	0
	*H*. *rustica*	B P	7	HIRUS17 (1) ^¥^	0	0
Laniidae	*Lanius minor*	B	1	0	0	0
Meropidae*	*Merops apiaster*	B P	1	0	0	0
Motacillidae	*Motacilla alba*	R W	4	YWT3 (1)	MOALB03 (1) ^¥^	0
Muscicapidae	*Ficedula hypoleuca*	P	1	0	0	0
	*F*. *semitorquata*	P B	4	FISEM02 (1) ^¥^ FISEM01 (1) ^¥^	0	0
	*Irania gutturalis*	B	1	0	0	0
	*Luscinia megarhynchos*	B	1	0	0	0
	*Oenanthe oenanthe*	P B	8	OEOEN01 (1) ^¥^	0	0
	*Saxicola torquata*	B	2	0	0	0
Passeridae	*Passer domesticus*	R	48	PADOM05 (27) PADOM32 (1) ^¥^ PAHIS2 (1)	GRW04 (1)	0
	*P*. *montanus*	R	36	PYERY01 (1) ^∞^	0	0
	*Petronia petronia*	B W R	7	PETPET02 (1) ^¥^ PETPET01 (1) ^¥^	0	0
Paridae	*Cyanistes caeruleus*	B	12	0	0	CYACAE06 (1) ^¥^
	*Parus major*	R	26	PARUS1 (2) ^∞^	0	0
Phylloscopidae	*Phylloscopus trochilus*	P	2	ACDUM1 (1) ^∞^	0	0
	*Seicercus nitidus*	P W B	1	0	ORW1(1)	0
	*S*. *trochiloides*	P	1	0	0	0
Sittidae	*Sitta tephronata*	R	4	PARUS1 (1) ^∞^ SITTEP01 (1) ^¥^	0	0
Sylviidae	*Curruca communis*	P B	2	0	0	0
	*Curruca curruca*	P	2	CUCUR01 (1) ^¥^	0	0
Sturnidae	*Sturnus vulgaris*	P W	5	LAMPUR01 (4)	0	0
Turdidae	*Turdus merula*	B W R P	15	TURDUS2 (6)	SYAT5 (1)	0
Upupidae *	*Upupa epops*	P B	1	0	0	0
Scolopacidae *	*Actitis hypoleucos*	P B W	1	0	0	0
Picidae *	*Dendrocopos syriacus*	R	5	SISKIN01 (1) ^∞^	0	0
Total			330	109 (33.03)	4 (1.21)	2 (0.6)

Non-passerine families (*), the frequent lineages in more than one species host (∞), and new lineages (¥) are specified.

The movement status of birds in Iran are shortened as P: passengers, B: breeding in summer, W: wintering, and R: resident.

### Microscopic inspection

To confirm the presence of blood parasites, 720 blood smears from all captured birds were examined. Due to the hemolysis of RBCs and reduced quality, a total of 37 slides were discarded from this study. Whole PCR positive specimens were matched with the morphological identification of parasite genera. A total of 23 bird species were identified to be harbored by *Haemoproteus*, and three and one species were infected by *Plasmodium* and *Leucocytozoon*, respectively.

### Haematoprotozoan molecular detection and infection rate

All collected blood samples were examined for haematoprotozoan infection and 115 individuals were positive for blood parasite genera using molecular approach (34.84% overall infection). Only one sample of *Emberiza buchanani* was co-infected with two genera of *Haemoproteus* and *Leucocytozoon*. The infection rates for *Haemoproteus*, *Plasmodium* and *Leucocytozoon* were 33.03%, 1.21% and 0.6% respectively (Table 1). Of the 43 avian species included in this study, 29 species (27 passerines and two non-passerines) harbored blood parasites. The bird species of *Iduna pallida* with four *Haemoproteus* lineages were the most frequently infected hosts by different parasites (Table 1).

### Lineages diversity

Based on morphological examination, repetitive PCR-positive samples of similar hosts in the same sampling localities were not sequenced. A total of 72 amplified samples by the nested PCR reactions were sequenced. Double peak sequences (co-infected) and poor-quality samples on the electropherograms were omitted from this study. Molecular identification of blood parasites using mtDNA *cytb* sequences detected 43 lineages of the collected bird samples from Iran. Lineages were attributed to the genera of *Haemoproteus* (n = 37), *Plasmodium* (n = 4), and *Leucocytozoon* (n = 2). Of these lineages, 20 were new, consisting of at least one base-pair difference from the lineages stored in MalAvi database (Table 1). Inspection of the found lineages revealed that only five *Haemoproteus* lineages (ACDUM1, ACDUM2, PARUS1, PYERY01, and SISKIN1) were infecting multiple hosts’ species (11.62%). Of which, three lineages were detected from different localities and PARUS1 was found in the same location of both hosts. Additionally, the lineages ACDUM1, ACDUM2, ACDUM3, ARW1, ACSTE1, BRW1, GRW03, HIP2, HIP4, LULU1, HIICT1, SISKIN01, CCF6, PYERY01, YWT3, PADOM05, PAHIS2, PARUS1, LAMPUR01, TURDUS2, GRW04, ORW1, and SYAT5 were 100 percent identical to the reported lineages in BLAST analyses (Table 1). The molecular approach could identify seven lineages of AEGCAU05, ALCATT01, CCF6, HIRUS17, YWT3, SYAT5, and EMBUC02 which were not recognized by the morphological method due to the presence of trophozoite or young gametocyte stages in prepared slides.

### *Haemoproteus*, *Plasmodium*, and *Leucocytozoon* novel lineages

The acquired results showed that 27 of the studied bird species were infected with *Haemoproteus* lineages of which 92.59% belonged to passerine hosts. *Haemoproteus* was the predominate genus in comparison with *Plasmodium* and *Leucocytozoon*. In this study, 17 new lineages of GRBRU01, CUCUR01, EMBUC01, FISEM01, FISEM02, GRMEL01, GRBRU02, HIRUS17, GRMEL02, PETPET01, OEOEN01, PETPET02, IDPAL01, SITTEP01, AEGCAU05, PADOM32, and ALCATT01 were detected from this region. Additionally, four avian species were infected with *Plasmodium* lineages, all of them being observed in passerines. MOALB03 was the only new lineage found in the collected samples. Moreover, in two infected hosts, novel *Leucocytozoon* lineages of CYACAE06 and EMBUC02 were observed in the passerine hosts (Table 1).

### Molecular phylogenetic analysis

The phylogenetic relationship of avian parasite genera was affirmed via attained Bayesian tree in three main clades using amplified sequences (n = 72) and retrieved MalAvi sequences (n = 95) are shown in Fig 1. For each genus, robust posterior probability (>0.8) supported for the main clades encompassed lineages collected from avian species in Iran. The largest number of lineages belonged to *Haemoproteus* with sub-clades not well supported for all lineages. The haemosporidians genera of *Haemoproteus* and *Plasmodium* appeared as sister groups and the *Leucocytozoon* clade was placed as the basal group. All *Haemoproteus* lineages were situated in one clade except for TURDUS2 (*Haemoproteus minutus*), HLW1, WW1, and COLL2 which was placed in a sister group.

## Discussion

As a study outlining the molecular detection of hematozoa of wild birds, the current study has recorded new lineages for three genera of *Haemoproteus*, *Plasmodium*, and *Leucocytozoon* from Iran. PCR-based molecular procedures for recognition of avian blood parasites provide a distinct chance to confirm numerous theories about their evolution, function, and specificity [31]. The relatively high frequency of newly identified lineages necessitates further studies in the Middle East region. In this study, results showed a total of 20 novel lineages and 23 previously recorded lineages within bird hosts from Iran (Table 1). Such findings will generate a database of host distribution and geographical range of hematozoan parasites [35]. Of the 72 amplified sequences of haemosporidians in this study, 53.48 percent of lineages have been identified by preceding studies around the world including ACDUM1, ACDUM2, ACDUM3, ARW1, ACSTE1, BRW1, GRW03, HIP2, HIP4, LULU1, HIICT1, SISKIN01, CCF6, YWT3, PADOM05, PAHIS2, PYERY01, PARUS1, LAMPUR01, TURDUS2, SYAT5, ORW1, and GRW04 (Table 2). Our results showed the new geographical locations of bird hosts for these listed lineages. In regards to the MalAvi database, 11 of the identified lineages were associated with pre-discovered morphospecies, *Haemoproteus belopolskyi* (ARW1 and HIICT), *H*. *pastoris* (LAMPUR01), *H*. *balmorali*)LULU1), *H*. *Passeris* (PADOM05), *H*. *majoris* (PARUS1), *H*. *tartakovskyi* (SISKIN01), *H*. *minutus* (TURDUS2), *H*. *motacillae* (YWT3), *Plasmodium relictum* (GRW04), and *P*. *vaughani* (SYAT5). Classification of parasites at species level and species delimitation in avian malaria parasites is very difficult, particularly in *Haemoproteus*, as a threshold of inter- and intra-species variation has not been set by the use of molecular markers [40]. This may explain the reason why most published articles in this field have focused on lineages instead of species level. There are more than 3132 identified unique lineages for avian malaria and the closely related genera from 1561 host species in MalAvi as of August 2018 [35].

**Table 2:** Detailed information about detected lineages, their previously identified hosts, new host records from Iran, related near lineages, and major hosts in some sampling locations is given.

Detected lineages	Previously identified hosts	Published locality	New hosts/ location from Iran	Related near lineage	Major host/ location
ACDUM1	*Acrocephalus dumetorum*	Lithuania [41]	*Acrocephalus dumetorum*/ S *Phylloscopus trochilus*/ E	*-*	*-*
ACDUM2	*Hippolais polyglotta* *Acrocephalus Agricola*	Spain, French, Germany [43] Bulgaria, Russia, Caucasia [44]	*Acrocephalus dumetorum*/ R S *Aegithalos caudatus*/ M	*-*	*-*
ACDUM3	*Acrocephalus dumetorum*	India [45]	*Acrocephalus dumetorum/* S	*-*	*-*
AEGCAU05 ^¥^	*-*	-	*Aegithalos caudatus*/ M	SISKIN01	*Loxia curvirostra*/ Lithuanaia and Russia [41,46], *Carduelis spinus/* Lithuania, Russia and Alaska [41,46], *Carpodacus mexicanus*/ New York, California [47,48], *Passer domesticus*/ California [49], *Carpodacus erythrinus/* Czech Republic [50], *Carduelis flammea*/ Alaska [51].
ALCATT01^¥^	*-*	-	*Alcedo atthis*/ M		
ARW1 (*H*. *belopolskyi*)	*Acrocephalus baeticatus*	Nigeria [52]	*Acrocephalus melanopogon*/ Z	*-*	*-*
ACSTE1	*Acrocephalus stentoreus* *Acrocephalus arundinaceus*	Lithuania [41] Romania, Bulgaria [53]	*Acrocephalus stentoreus*/ R	*-*	*-*
BRW1	*Acrocephalus griseldis*	Kenya [5]	*Acrocephalus stentoreus*/ R	*-*	*-*
CYACAE06 ^¥^	*-*	-	*Cyanistes caeruleus/* M	PARUS84	*Cyanistes caeruleus*/ Portugal [54]
CUCUR01 ^¥^	*-*	-	*Curruca curruca*/ S	CWT04	*Acrocephalus palustris/* Russia [19]
EMBUC01 ^¥^	*-*	-	*Emberiza buchanani/* Z	EMSPO01	*Emberiza spodocephala/* Russia [46]
EMBUC02 ^¥^	*-*	-	*Emberiza buchanani*/ Z	ROFI6	*Carpodacus erythrinus/* Czech Republic [55], *Carduelis flammea/* Alaska [51]
FISEM01 ^¥^	*-*	-	*Ficedula semitorquata*/ R	ESTMEL01	*Estrilda melanotis*/ Tanzania [55]
FISEM02 ^¥^	*-*	-	*Ficedula semitorquata/* Z	FOUMAD01	*Foudia madagascariensis/* Madagascar [55]
GRBRU01 ^¥^	*-*	-	*Granativora bruniceps* / G	ALARV03	*Alauda arvensis*/ Italy [56]
GRBRU02 ^¥^	*-*	-	*Granativora bruniceps* / S	PADOM22	*Passer domesticus/* Spain [49]
GRMEL01 ^¥^	*-*	-	*Granativora melanocephala/* Z	JUHYE03	*Junco hyemalis/* Alaska [51]
GRMEL02 ^¥^	*-*	-	*Granativora melanocephala/* Z	PAGRI04	*Passer griseus/* Kenya [55]
GRW03	*Acrocephalus arundinaceus*	Kenya [5], Bulgaria [41,53]	*Acrocephalus stentoreus*/ K R	*-*	*-*
HIP2	*Hippolais pallida* *Hippolais caligata*	Nigeria [52] Russia [46]	*Iduna pallida*/ Z	*-*	*-*
HIP4	*Hippolais pallida*	Nigeria [52]	*Iduna pallida*/ S	*-*	*-*
HIRUS17 ^¥^			*Hirundo rustica*/ R	PAGRI01	*Passer griseus*/ Nigeria (Bensch & Ottosson unpubl) refrenced by MalAvi [35]
LULU1 (*H*. *balmorali*)	*Luscinia luscinia*	Russia [57]	*Iduna pallida*/ S	*-*	*-*
HIICT1 (*H*. *belopolskyi*)	*Hippolais icterina* *Hippolais polyglotta* *Saxicola rubetra* *Sylvia borin* *Acrocephalus agricola*	French [43], Russia [58] Germany [43] Nigeria [41] Nigeria [41] Bulgaria [53]	*Hippolais polyglotta*/ Z	*-*	*-*
IDPAL01 ^¥^	*-*	-	*Iduna pallida/* G	RW2	*Acrocephalus scirpaceus/* Nigeria [52], *Acrocephalus arundinaceus*/ Sweden [59], *Iduna opaca*/ Morocco [54]
OEOEN01 ^¥^	*-*	-	*Oenanthe oenanthe*/ E	PAMEL01	*Passer melanurus*, *Passer diffuses*, *Hirundo abyssinica*/ Africa [55]
PETPET01 ^¥^	*-*	-	*Petronia petronia*/ S	PAGRI04	*Passer griseus/* Kenya [55]
PETPET02 ^¥^	*-*	-	*Petronia petronia*/ Z	ROFI1	*Fringilla coelebs*/ Russia [19], *Carduelis chloris*, *Carpodacus erythrinus* / Sweden [41]
PADOM32 ^¥^	*-*	-	*Passer domesticus/* G	VILWE3	*Ploceus cucullatus/* Nigeria, *Ploceus nigerrimus*, *Ploceus velatus*/ Gabon and Nigeria [41]
SITTEP01 ^¥^	*-*	-	*Sitta tephronata/* Z	SITEUR01	*Sitta europaea*/ Morocco [54]
MOALB03 ^¥^	*-*	-	*Motacilla alba*/ M.	GEOTRI02	*Geothlypis trichas* [7], *Passerina amoena*/ America [48].
SISKIN01 (*H*. *tartakovskyi*)	*Carduelis flammea*, *Carduelis spinus*	Alaska [51]	*Carduelis carduelis*/ S-R *Dendrocopos syriacus*/ Z	*-*	*-*
CCF6	*Fringilla coelebs* *Cyanistes caeruleus*	Russia [60], Bulgaria [61], Sweden [41], Morocco, Portuguese, Azerbaijan, Armenia [60] Morocco, Portuguese [54,60]	*Fringilla coelebs*/ R	*-*	*-*
YWT3 (*H*. *motacillae*)	*Motacilla flava*	Spain [43]	*Motacilla alba/* E	*-*	*-*
PADOM05 (*H*. *passeris*)	*Passer moabiticus* *P*. *domesticus* *Sylvia borin*	Palestine [62] French, Turkey, Russia, Spain [49,63] Spain [64]	*Passer domesticus*/ Z-A-S-R-G-K	*-*	*-*
PAHIS2	*Passer hispaniolensis*	Morocco [54]	*Passer domesticus*/ R	*-*	*-*
PYERY01	*Pyrrhula erythaca* *Pyrrhula pyrrhula*, *Carpodacus erythrinus*, *Serinus striolatus*	California [65] Russia [46]	*Linaria cannabina*/ A *Passer montanus*/ R	*-*	*-*
PARUS1 (*H*. *majoris*)	*Parus major* *Panurus biarmicus*, *Hippolais icterina*, *Ficedula hypole*, *Sitta europaea*, *Emberiza schoeniclus*	Russia [19] Sweden [41]	*Parus major* & *Sitta tephronata*/ S	*-*	*-*
LAMPUR01 (*H*. *pastoris*)	*Lamprotornis purpureiceps* *Sturnus roseus* *S*. *vulgaris*	Gabon [66] Bulgaria [67] Bulgaria [67]	*Sturnus vulgaris*/ S	*-*	*-*
TURDUS2 (*H*. *minutus*)	*Turdus philomelos* *T*. *merula*	Sweden [52] Sweden [40,41]	*Turdus merula*/ S	*-*	*-*
SYAT05 (*P*. *vaughani*)	*Sylvia atricapilla* *Turdus merula*	Italy [41] French, new Zealand [68]	*Turdus merula*/ S	*-*	*-*
ORW1	*Acrocephalus orientalis* *Phylloscopus trochilus* *Ph*. *trochiloides*	Japan [5] England [5] Russia, Caucasia [69]	*Seicercus nitidus*/ S	*-*	*-*
GRW04 (*P*. *relictum*)	*Acrocephalus arundinaceus* *Passer domesticus*	Nigeria [52] America, Bermuda, India [7]	*Passer domesticus*/ R	*-*	*-*

Provinces of sampling are abbreviated by A (Ardabil), Zanjan (Z), Semnan (E), North Khorasan (S), Razavi Khorasan (R), Golestan (G), Mazandaran (M), and Gilan (K).

New lineage are specified by (^¥^).

The new amplified sequences of this study are illustrated in the phylogenetic tree with the nearest lineages obtained from BLAST analysis and prevoiusly reported lineages of similar hosts (Fig 1). All sequences were clustered in three main monophyletic clades as *Haemoproteus*, *Plasmodium*, and *Leucocytozoon* lineages. Two new lineages of *Leucocytozoon* are clustered as sister groups in two sub-clades of clade A. Likewise, the *Plasmodium* lineages in clade C are grouped as the sister taxa of *Haemoproteus* lineages in clade D.

Nucleotide distinctiveness collation with previously recorded sequences confirmed the relative shift of *Haemoproteus* lineages among birds found in Iran. Five shared lineages of ACDUM1, ACDUM2, PARUS1, PYERY01, and SISKIN1 were detected in different hosts from dissimilar families. ACDUM1 were observed in *Acrocephalus dumetorum* from North Khorasan and *Phylloscopus trochilus* from Semnan. ACDUM2 were collected in both *Acrocephalus dumetorum* from Razavi Khorasan & North Khorasan and *Aegithalos caudatus* form Mazandaran. Both infected hosts of PARUS1 (*Parus major* & *Sitta tephronata*) were recorded from North Khorasan. The species *Passer montanus* from Razavi Khorasan & *Linaria cannabina* from Ardabil were infected by PYERY01. Lineage of SISKIN1 were found in *Carduelis carduelis* from Razavi Khorasan and North Khorasan and non-passerine species of *Dendrocopos syriacus* from Zanjan province. Altogether these common lineages belong to *Haemoproteus* spp. (Table 2). Recent studies on blood parasites have recorded numerous lineages listed in Table 2 with detailed information about sampling locality in comparison with collected samples as new distribution and hosts from Iran. There is increasing evidence that haemosporidian infections in non-competent hosts result in abortive development of the parasites before they reach the stage of infectious gametocytes. As such DNA may leak into the blood, it is possible that the host range across bird taxa is an overestimation of parasite distribution within their competant hosts. Thus, microscopic examination of blood films remains a gold complementary method in the field of haemosporidian parasite studies [41].

Moreover, considering that most of the investigated birds do not inhabited and have entered Iran through migration routes, detection of more than half of the common previously identified lineages from another part of the world could be a reason for this (Table 1). Iranian avifauna is influenced by three birds’ migration routes, consisting of Central Asian Flyway, West Asian/East African Flyway, and Black sea/Mediterranean Flyway. Of the examined birds, the species of *Acrocephalus dumetorum*, *A*. *melanopogone*, *Aegithalos caudatus*, *Galerida cristata*, *Carduelis carduelis*, *Linaria cannabina*, *Serinus pusillus*, *Motacilla alba*, *Ficedula hypoleuca*, *Passer domesticus*, *P*. *montanus*, *Petronia petronia*, *Parus major*, *Sitta tephronata*, *Turdus merula*, and *Dendrocopos syriacusi* are resident in Iran while the rest are passengers, wintering or breeding in the summer [42].

Due to the lower divergence rate in malarial parasites rather than vertebrates, very close lineages can be found in different hosts of various families [6]. For instance, SISKIN1 and AEGCAU05 with 0.4% genetic variation were detected in two hosts of Fringillidae and Aegithalidae with high genetic differences. Species of *Haemoproteus* are often considered to be specific to birds within a family or subfamily [70,71]. However, there is experimental evidence which shows the successful transmission of the hemoproteids between birds belonging to different families/sub-families of the same order. It remains unclear how often such host shifts occur in wildlife. Fallon *et al*. (2003, 2005) provided the first molecular evidence that the same lineages of *Haemoproteus* spp. are present in birds belonging to different families of Passeriformes [72,73]. Szymanski and Lovette (2005) showed that *Haemoproteus* lineages recovered from two or more host individuals were found in at least two host families. This data indicates that some *Haemoproteus* spp. lineages exhibit a low degree of host specificity [8].

Haemosporidians as the most recurrent blood parasites, showed the comparatively high infection rate of 34.83% in the present study. In another investigation, the reported infection rate of avian hosts around the world were 29.5% in China [74], 12% in tropical regions of Costa Rica [75], 33% in Philippines [76], 35% in neotropical areas in Brazil [77], 40% in central Africa [6], and 50% in India [78]. *Haemoproteus* was the most affluent in comparison with other genera from the northern part of Iran which was in accordance with preceding studies [13,74,76]. In recent studies, the lowest recorded overall prevalence of *Haemoproteus*, *Plasmodium*, and *Leucocytozoon* was 3.1% and eight new lineages were found in South American waterfowls (from Peru and Argentina) [15]. The relatively medium prevalence of haemosporidians in the collected samples from Peninsular Malaysia was 30.3% and *Haemoproteus* were the predominant genus in comparison with *Plasmodium*. Moreover, 10 new lineages of *Haemoproteus* spp. and three new lineages of *Plasmodium* spp. were reported by molecular technique using *cytb* gene [16]. Very high overall infection prevalence of 83.6% was registered for two sympatric sand hill crane populations seized in Texas [79]. Likewise, another investigation on captive birds from a Brazilian megalopolis, have reported the overall prevalence 97.6% of the genus *Plasmodium* and 2.4% of *Haemoproteus* and 14 new lineages of *Plasmodium* spp. and two lineages of *Haemoproteus* spp. were detected using mitochondrial gene of *cytb* [80]. This amount of difference may be related to the lower pathogenicity in regard to *Plasmodium*. Infected birds can rarely be captured by mist net due to their lessened mobility and activity and also due to the variation in vector abundance [1]. Additionally, several factors such as temporal and spatial influence, gender, age, immunological system, distinctive study approaches and various collected taxa may influence the prevalence rate in hematozoan parasites [15,16,81]. As the collection of samples for potential vectors in this study were performed near aquatic localities and rivers, the infection rate by *Plasmodium* and *Leucocytozoon* parasites were recorded as very low in contrast with *Haemoproteus* sampled throughout Iran, which may be attributed to the lack of available vectors in these regions.

The molecular and morphological techniques are complementary to each other. In our study a molecular approach was successful in the identification of seven lineages AEGCAU05, ALCATT01, CCF6, HIRUS17, YWT3, SYAT5, and EMBUC02 which failed to be identified through inspection of prepared slides from birds with a low level of infection [82]. Findings of similar studies also demonstrated the effectiveness of more sensitive PCR-based methods in comparison with the morphological detection of apicomplexan blood parasites [19,78]. Moreover, other investigations have demonstrated an equal efficiency of both methods of blood parasite discovery [16].

In this study, our results demonstrated that Iran as a corridor in birds migratory routes may be influenced to have the high risk of exposure to new parasites. Thereupon, previously common identified lineages may be detected in new hosts in unchecked regions (e.g. more than 50% of our detected lineages). As the molecular study for detection of birds hematozoa from Iran, 20 novel lineages for three genera of *Haemoproteus*, *Plasmodium*, and *Leucocytozoon* were reported from this region and about 12% of lineages of were observed in multiple species from different families of birds. Additional investigations into these taxa in the avifauna for other parts of Iran may attain further data on the detection of new lineages, and pursue the relationship and distribution pattern between blood parasites and hosts.

## Supporting information

S1 Table The exact coordinate of sampling site of each province from Iran. (DOC)
