# Correction: Molecular detection and genetic diversity of avian haemosporidian parasites in Iran

**DOI:** 10.1371/journal.pone.0212453

**Published:** 2019-02-11

**Authors:** Leila Nourani, Mansour Aliabadian, Omid Mirshamsi, Navid Dinparast Djadid

The following information is missing from the Funding section: The authors would like to thank the Iran National Science Foundation for the financial support of this research project (93035204).

The Data Availability statement for this paper is incorrect. The correct statement is: The amplified sequences of the parasite lineages (mitochondrial cytb gene) were deposited in GenBank (Accession numbers MG976505-MG976576). More data about sampling and hosts and sequences are deposited in MalAvi data center (http://mbio-serv2.mbioekol.lu.se/Malavi/).

The authors would like to provide a .png version of Fig 1 in their Supporting Information. Please see S1 Fig below.

## Supporting information

S1 Fig Bayesian phylogenetic tree of blood parasites of mitochondrial DNA *cytb* lineages attained from infected Iranian birds and MalAvi sequences. Posterior probability values (>0.8) are given. Provinces of sampling are abbreviated by Ardabil (A), Zanjan (Z), Semnan (E), North Khorasan (S), Razavi Khorasan (R), Golestan (G), Mazandaran (M) and Gilan (K). Schematic images of birds were retrieved from www.HBW.com .
(PNG)
