# Supplementary material for: Molecular detection and genetic diversity of avian haemosporidian parasites in Iran
Source: PLoS One. 2018 Nov 9;13(11):e0206638. doi: 10.1371/journal.pone.0206638 (PMC6226148; doi:10.1371/journal.pone.0206638)
Supplement: S1 Table — (DOC) [file pone.0206638.s001.doc]

**S1 Table.**

| **Provinces** | **Sampling site** | **Longitude** | **Latitude** |
| --- | --- | --- | --- |
| **Ardabil** | Takhte soleiman | 48˚ 39' 71.8'' | 37˚ 88' 67.2'' |
|  | Amirabad | 48˚ 35' 66.7'' | 37˚ 83' 94.8'' |
| **Golestan** | Dasht | 56˚ 01' 05.7'' | 37˚ 17' 23.5'' |
|  | Golestan | 56˚ 01' 28.1'' | 37˚ 19' 46.5'' |
|  | Mahgol | 55˚ 43' 03.5'' | 37˚ 23' 25.7'' |
|  | Kurdkuy | 54˚ 7' 18.3'' | 36˚ 47' 34.1'' |
|  | Aliabad | 54˚ 51' 48.4'' | 36˚ 54' 29.2 |
| **Gilan** | Anzali | 49˚ 46' 67'' | 37˚ 45' 69'' |
|  | Anzali | 48˚ 32' 11.09'' | 37˚ 23' 60.3'' |
|  | Ghachsar | 49˚ 2' 35'' | 37˚ 39' 25.2'' |
| **Mazandaran** | Noor | 52˚ 02' 45.3'' | 36˚ 34' 54.8'' |
|  | Rooyan | 51˚ 59' 20.9'' | 36˚ 32' 03.2'' |
|  | Amol | 52˚ 38' 24.7'' | 36˚ 53' 22.1'' |
|  | Sari | 53˚ 12' 19.9'' | 36˚ 51' 068'' |
| **North Khorasan** | Zartanloo | 58˚ 08' 23'' | 37˚ 31' 33.4'' |
|  | Zu | 57˚ 45' 29'' | 37˚ 43' 44.2'' |
|  | Sade shirvan | 57˚ 57' 30.5'' | 37˚ 36' 19.7'' |
|  | Kharkhane siman | 57˚ 41' 56.1'' | 37˚ 23' 17.1'' |
|  | Ghare bashloo | 57˚ 17' 07.3'' | 37˚ 22' 31.11'' |
|  | Dargaz | 58˚ 33' 16.9'' | 37˚ 30' 18.4'' |
| **Razavi Khorasan** | Moghan | 59˚ 36' 66.8'' | 36˚ 13' 22.2'' |
|  | Bazangan Lake | 60˚ 26' 34'' | 36˚ 18' 29.3'' |
|  | Baghan | 57˚ 76' 98.9'' | 36˚ 19' 43.6'' |
|  | Somea | 58˚ 84' 93.2'' | 36˚ 27' 91'' |
|  | Boozhan | 58˚ 96' 94.6'' | 36˚ 23' 64.4'' |
|  | Abardeh | 59˚ 27' 50.2'' | 36˚ 38' 53'' |
|  | Kalat | 59˚ 74' 57.1'' | 37˚ 00' 0.25'' |
|  | Mashhad | 59˚ 53' 062'' | 36˚ 30' 92.9'' |
| **Semnan** | Negarman | 54˚ 49' 09.0" | 36˚ 31' 57.2" |
|  | Mojen | 54˚ 38'54.2" | 36˚ 28' 52.1" |
| **Zanjan** | Gharanas | 47˚ 95' 0.17'' | 37˚ 49' 87.7'' |
|  | Khorjahan | 48˚ 07' 034'' | 37˚ 34' 44.5'' |
|  | Dandi | 48˚ 29' 24'' | 37˚ 26' 30'' |
