# Supplementary figures and images for: Correction: Molecular detection and genetic diversity of avian haemosporidian parasites in Iran
Source: PLoS One. 2019 Feb 11;14(2):e0212453. doi: 10.1371/journal.pone.0212453 (PMC6370224; doi:10.1371/journal.pone.0212453)

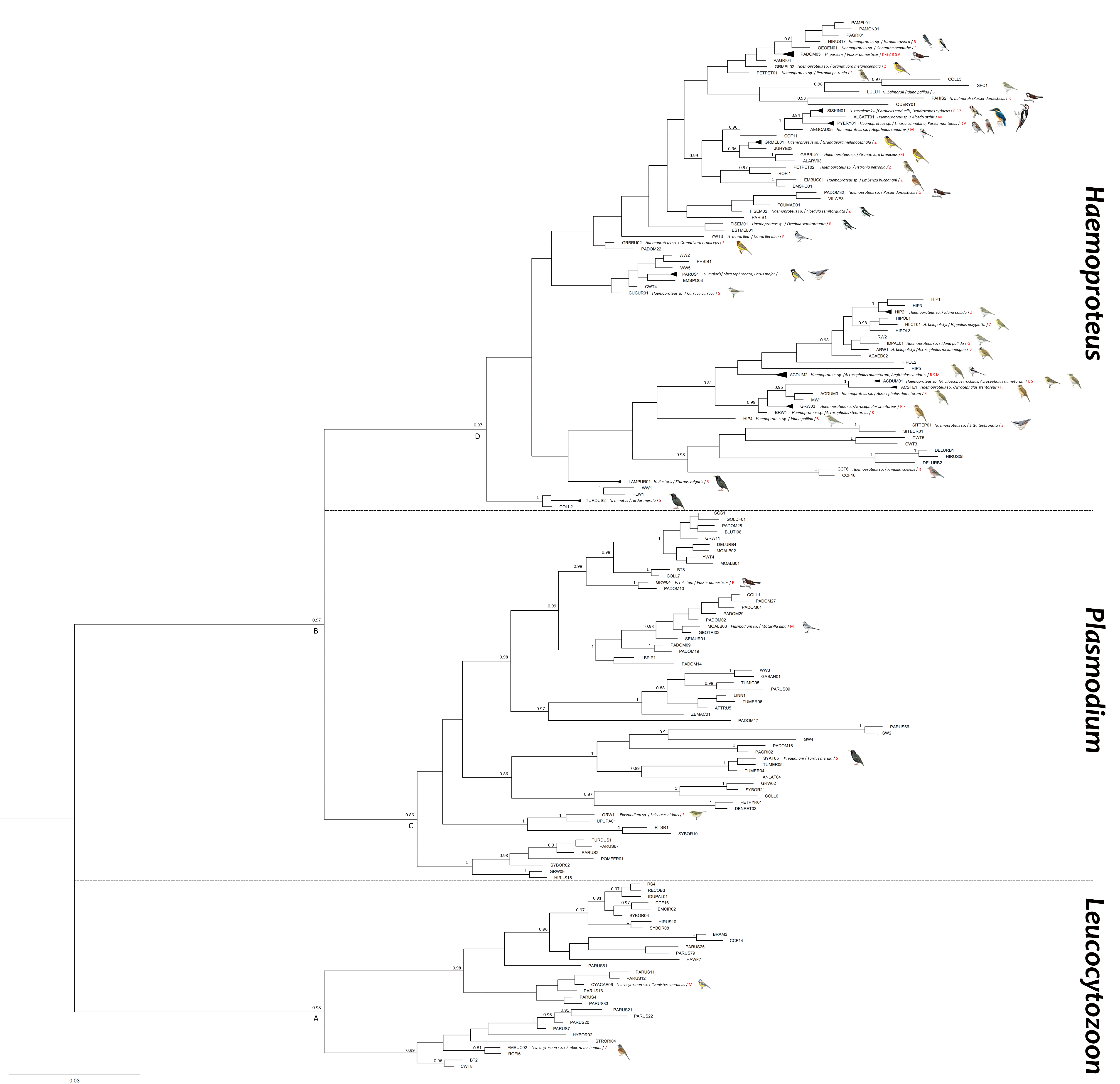

Supplement: S1 Fig — Posterior probability values (>0.8) are given. Provinces of sampling are abbreviated by Ardabil (A), Zanjan (Z), Semnan (E), North Khorasan (S), Razavi Khorasan (R), Golestan (G), Mazandaran (M) and Gilan (K). Schematic images of birds were retrieved from www.HBW.com . (PNG) [file pone.0212453.s001.png]
